# Synthesis and characterization of CuO nanowires by a simple wet chemical method

**DOI:** 10.1186/1556-276X-7-70

**Published:** 2012-01-05

**Authors:** Anita Sagadevan Ethiraj, Dae Joon Kang

**Affiliations:** 1 BK21 Physics Research Division, Department of Energy Science, Institute of Basic Science, Sungkyunkwan University, 300 Cheoncheon-dong, Jangan-gu, Suwon, 440-746, South Korea

## Abstract

We report a successful synthesis of copper oxide nanowires with an average diameter of 90 nm and lengths of several micrometers by using a simple and inexpensive wet chemical method. The CuO nanowires prepared via this method are advantageous for industrial applications which require mass production and low thermal budget technique. It is found that the concentration and the quantity of precursors are the critical factors for obtaining the desired one-dimensional morphology. Field emission scanning electron microscopy images indicate the influence of thioglycerol on the dispersity of the prepared CuO nanowires possibly due to the stabilization effect of the surface caused by the organic molecule thioglycerol. The Fourier transform infrared spectrum analysis, energy dispersive X-ray analysis, X-ray diffraction analysis, and X-ray photoemission spectrum analysis confirm clearly the formation of a pure phase high-quality CuO with monoclinic crystal structure.

## Introduction

Since the discovery of carbon nanotubes, the synthesis of one-dimensional morphology such as nanowires and nanorods has gained much attention because this constitutes an important building block of nanodevices and integrated nanosystems [1-5]. Among the available transition metal oxides, such as Ni, Cu, Zn, and Fe, synthesis of CuO is an important topic of research. Cupric oxide, CuO, which is a p-type semiconductor [6,7] (indirect bandgap of 1.2 to 1.5 eV) has been widely exploited for diverse applications, such as an active electrode material for Li-ion batteries, field emission [FE] emitters, heterogeneous catalysts, gas sensors, and solar cells [8-13]. Moreover, the evidence of a spin-dependent quantum transport phenomenon in CuO nanowires was already reported [14]. Till now, many methods have been developed to synthesize CuO nanowires or nanorods, such as thermal oxidation of copper foil, hydrothermal route, aqueous reaction, vapor-liquid-solid synthesis, solution-liquid-solid synthesis, laser ablation, arc discharge, precursor thermal decomposition, electron beam lithography, and template-assisted synthesis [5,15-19]. However, all these methods either require high temperatures, sophisticated instrumentation, inert atmosphere, or long reaction time. The difference between the method in this manuscript and the aqueous reaction referred earlier is the starting precursor material used and the stabilizer. In our case, the precursor used is copper acetate, while in the aqueous reaction, copper chloride. We used the organic molecule thioglycerol [TG] as stabilizer, while no stabilizer was used in the latter case.

Moreover, until now, few reports are available in literature for the synthesis of CuO nanowires using the organic molecule TG. Therefore, in the present study, a systematic effort has been made to synthesize CuO nanowires by a simple and inexpensive wet chemical method using copper acetate and NaOH as the precursor material in the presence of organic molecule TG. The possible formation mechanism of CuO nanowires via this chemical method is also discussed.

## Experimental detail

All the reagents were of analytical grade and were used without further purification. Copper acetate [(CH_3_COO)_2_-H_2_O] and sodium hydroxide [NaOH] were used as precursors in the present experiment. Two separate solutions, copper acetate (0.5 M) in deionized [DI] water and NaOH (5 M) in DI water, were prepared. Aqueous copper acetate and aqueous NaOH solutions were referred to as solution A and solution B, respectively. Stirring is continued until the respective metal salts are completely dissolved in DI water. Later 1 μL of TG is added to solution A, and the solution is stirred for a few minutes. Solution B is then added to the reaction mixture, and water is immediately added. Further stirring continued for a few minutes. Centrifugation is done to collect the precipitate. Washing of the precipitate is carried out using the DI water for five to six times. Finally, the collected precipitate is dried overnight at 35°C.

The morphology of the CuO nanowires obtained in the present work was investigated by a field emission scanning electron microscope [FE-SEM] (JEOL JSM-7401F; JEOL Ltd., Akishima, Tokyo, Japan) operated at an accelerated voltage of 10 kV. The energy dispersive X-ray analysis [EDX] was carried out on the scanning electron microscopy [SEM] system. X-ray diffraction [XRD] spectra of the CuO samples were obtained using a powder X-ray diffractometer (D8 FOCUS 2200 V Bruker AXS, Bruker Optik Gmbh, Ettlingen, Germany), using Cu K*α *radiation (*λ *= 1.5418 Å) with 2*θ *ranging from 20° to 80°. The Fourier transform infrared spectrum [FTIR] of CuO samples in the form of pellets was recorded using a Perkin Elmer 1615 spectrometer (PerkinElmer, Waltham, MA, USA). Pellets were prepared by mixing CuO powder with KBr, and spectrum was recorded in the range of 400 to 4,000 cm^-1^. X-ray photoelectron spectroscopy [XPS] measurements were performed on ESCA MK II (VG Scientific Ltd., London, England) set up by using an Al Kα X-ray source (*hν *= 1486.6 eV). All the experiments were carried out at a base pressure of approximately 10^-9 ^mbar, and a C 1*s *spectrum at 285.0 ± 0.2 eV served as the internal reference.

## Results and discussion

**Figure 1:**
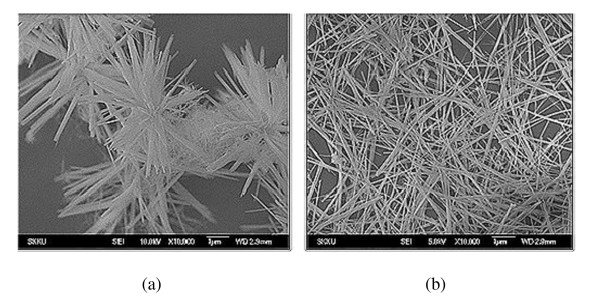
**SEM micrograph for the CuO nanowires prepared (a) without TG and (b) with TG**.

CuO nanowires were synthesized by a wet chemistry route in the presence of organic molecule TG. Figure 1a represents the FE-SEM image of the as-synthesized CuO nanowires without TG, and Figure 1b, in the presence of TG. The morphology of the CuO samples without TG shows the formation of CuO flowers consisting of individual nanowires, whereas when the same synthesis is carried out in the presence of organic molecules TG, isolated CuO nanowires were obtained. Thus, the presence of a small amount of TG can render the nanowires of CuO well-dispersed, as seen clearly in the micrograph of CuO with TG. The average diameter of the CuO nanowires was observed to be around 90 nm with a length of about 2 to 5 μm.

In the synthesis of CuO nanowires, when copper acetate reacted with sodium hydroxide in the aqueous medium, the following reaction takes place as stated in Equation 1. This particular reaction does not involve any templates or substrates or any structure-directing agent like cetyltrimethylammonium-bromide [CTAB] or hexamethylene tetramine [HMTA] [20,21]. However, we introduced the organic molecule TG to the copper acetate solution before reacting with NaOH. In the present synthesis, the concentration of Cu(OAc)_2 _and NaOH and the reaction time are the critical parameters to obtain the nanowire morphology. Since the surface passivation of quantum dots using TG is well documented in literature [22,23], we have tried to utilize for the very first time the same organic molecule TG in the synthesis of CuO. From the SEM results, we observe that when no TG was used, we obtained CuO flowers consisting of individual nanowires. This result is in good agreement with the work reported by Zhu et al [24,25].

(1) $$\text{CuC}{\text{H}}_{\text{3}}{\left(\text{COO}\right)}_{\text{2}}\cdot {\text{H}}_{\text{2}}\text{O }+\text{ NaOH}\overset{\text{TG}}{\underset{}{\to }}\text{CuO }+\text{ 2Na}\left(\text{C}{\text{H}}_{\text{3}}\text{COO}\right)\;+\;{\text{H}}_{\text{2}}\text{O}$$

When the reaction in Equation 1 proceeds with the nucleation and crystal growth, the Gibbs free energy of the nanocrystallite surfaces is very high, and in order to decrease the Gibbs free energy, the nanocrystallites tend to aggregate [26]. Therefore, the flower morphology of CuO is obtained. However, when a small amount of TG is introduced in the synthesis of CuO, instead of a flower morphology, well-dispersed CuO nanowires are obtained, which can be speculated due to the stabilization effect caused by the use of organic molecule TG. Moreover, the role of TG is different from what structure-directing agents such as CTAB and HMTA are doing. In our case, without any structure-directing agent, we are able to generate CuO nanoflowers. When we carried out the CuO synthesis using TG, we obtained only separated CuO nanowires which clearly imply that the role of TG is definitely different from the role of CTAB and HMTA, where use of these agents leads to some kind of morphological changes in the material formation. Hence, the role of TG in our case is just for dispersion due to surface stabilization. An in-depth study is required, and further experiments are underway to understand and investigate the exact mechanism and the role of TG in the synthesis of CuO nanowires.

In order to detect the elements present in the CuO samples, EDX analysis was carried out. The spectrum (not shown) indicates the presence of copper [Cu], oxygen [O], and silicon [Si]. No other impurity was detected. The presence of Si was from the substrate. Thus, the formation of CuO was confirmed from the EDX.

**Figure 2:**
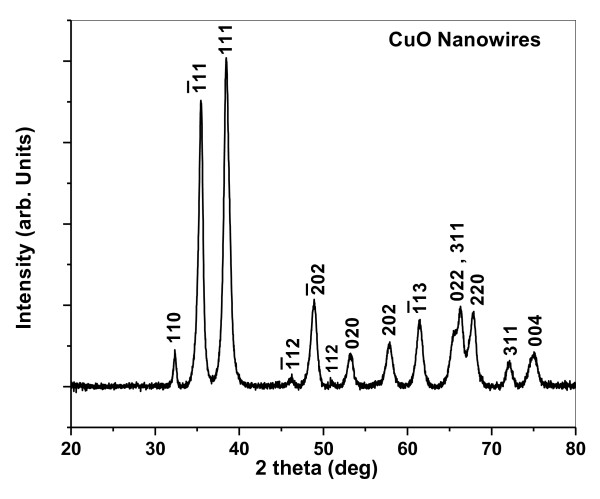
**XRD pattern of the as-synthesized CuO nanowires with TG**.

The X-ray diffraction pattern of the CuO samples prepared in the presence of TG is depicted in Figure 2. It can be clearly seen that all the peaks in the XRD patterns are consistent with the JCPDS data (48-1548) of the CuO with a monoclinic phase. No characteristic peaks of any other impurities such as Cu(OH)_2_, Cu_2_O, or precursors used are observed, indicating the formation of a pure phase CuO.

**Figure 3:**
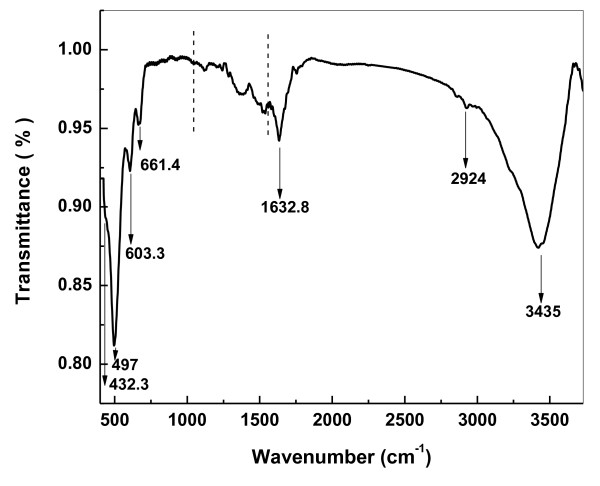
**The FTIR spectrum of the as-synthesized CuO nanowires in the presence of TG**.

**Figure 4:**
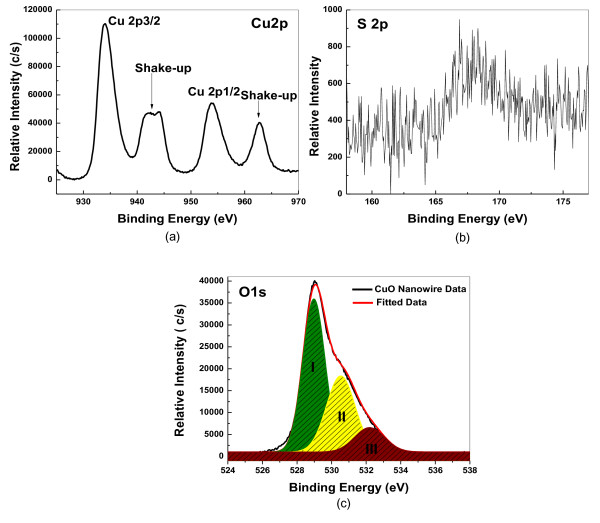
**X-ray photoelectron spectra**. (**a**) Cu 2*p*, (**b**) S 2*p*, and (**c**) O 1*s *deconvolution of the CuO nanowires synthesized with TG.

In order to understand the chemical and structural nature of the synthesized CuO and the effect of the chemicals used in the synthesis of CuO nanowires, FTIR analysis was carried out. Figure 3 represents the FTIR spectrum recorded for the CuO nanowires in the range of 400 to 4,000 cm^-1^. The three characteristic bands observed at 432.3 cm^-1^, 497 cm^-1^, and 603.3 cm^-1 ^can be assigned to the Au mode, Bu mode, and the other Bu mode of CuO [27]. The high-frequency mode at 603.3 cm^-1 ^may be attributed to the Cu-O stretching along the [101] direction, while the peak at 497 cm^-1 ^can be assigned to the Cu-O stretching vibration along the [101] direction [28]. Moreover, no other IR active mode was observed in the range of 605 to 660 cm^-1^, which totally rules out the existence of another phase, i.e., Cu_2_O [29]. Moreover, the C-S bond observed at 661.4 cm^-1 ^can be attributed to the organic molecule TG used in the synthesis of CuO nanowires. Thus, the pure phase CuO with monoclinic structure is also confirmed from the FTIR analysis. The elemental composition and oxidation states of the CuO samples were analyzed using XPS. The survey scan recorded (not shown) for the sample shows the presence of Cu and O. A trace amount of sulfur which is detected comes from the TG. A selected area scan is also recorded for the individual elements (Cu 2*p*, S 2*p*, and O 1*s*) and is shown in Figure 4a, b, c, respectively. The Cu 2*p *core-level spectrum (Figure 4a) represents two peaks located at 934 and 953.8 eV which corresponds to the Cu 2*p*3/2 and Cu 2*p*1/2, respectively. These values match well with the data reported for the Cu(2*p*) in CuO [18,30-32]. Also, the width of approximately 19.8 eV between these two Cu peaks is the same as in the standard spectrum of Cu. In addition, the shake-up satellite peaks located at 942.7 eV and 944.8 eV are at a higher binding energy value of 8.8 and 10.1 eV, respectively, when compared with the main sharp peak of Cu 2*p*3/2 located at 934 eV [30]. The strong shake-up satellites recorded in the CuO sample confirm the Cu(II) oxidation state and rule out the possibility of the existence of a Cu_2_O phase [18,32]. The O 1*s *core level spectrum is shown in Figure 4c, which demonstrates a broad Gaussian peak and is deconvulated as peaks I, II, and III, respectively. The peak I at the low binding energy value of 529 eV is due to the oxygen in the CuO crystal lattice, which corresponds to the O-Cu bond, whereas the peaks II and III located at the higher binding energy values of 530.5 eV and 532.3 eV are due to the chemisorbed oxygen caused by surface hydroxyl groups which are associated with the O-H bond [18]. From the elemental scan of sulfur (Figure 4b), one can see that a small amount of sulfur detected in XPS indicates the presence of TG on the surface of the CuO nanowires synthesized.

## Conclusions

Using a simple and inexpensive wet chemical method, the synthesis of copper oxide nanowires with diameters of 90 nm and lengths of several micrometers has been successfully carried out. The concentration and quantity of precursors are the critical factors for obtaining the desired 1-D morphology. SEM micrographs clearly indicate the influence of TG on the dispersity of the prepared CuO nanowires which may be due to the stabilization effect of the surface caused by the organic molecule TG. The FTIR and XPS data analyses confirm the formation of a pure phase CuO with monoclinic crystal structure. EDX and XRD data support the same finding.

## Competing interests

The authors declare that they have no competing interests.

## Authors' contributions

ASE did the synthesis and performed tests on the samples. DJK conceived and designed the experiments. ASE and DJK wrote the manuscript. All authors read and approved the final manuscript.
